# Recent Advances in Nanomaterials for Gene Delivery—A Review

**DOI:** 10.3390/nano7050094

**Published:** 2017-04-28

**Authors:** Michael K. Riley, Wilfred Vermerris

**Affiliations:** 1 Graduate Program in Plant Cellular and Molecular Biology, University of Florida, Gainesville, FL 32611, USA; mike.riley2@ufl.edu; 2 UF Genetics Institute, University of Florida, Gainesville, FL 32611, USA; 3 Department of Microbiology & Cell Science, University of Florida, Cancer/Genetics Research Complex 302, 2033 Mowry Road, Gainesville, FL 32610, USA

**Keywords:** gene delivery, nanomaterials, non-viral vectors, siRNA

## Abstract

With the rapid development of nanotechnology in the recent decade, novel DNA and RNA delivery systems for gene therapy have become available that can be used instead of viral vectors. These non-viral vectors can be made of a variety of materials, including inorganic nanoparticles, carbon nanotubes, liposomes, protein and peptide-based nanoparticles, as well as nanoscale polymeric materials. They have as advantages over viral vectors a decreased immune response, and additionally offer flexibility in design, allowing them to be functionalized and targeted to specific sites in a biological system with low cytotoxicity. The focus of this review is to provide an overview of novel nanotechnology-based methods to deliver DNA and small interfering RNAs into biological systems.

## 1. Introduction

Nanotechnology has seen many advances in recent years, both in terms of the development of novel materials, often with tunable properties [1,2] and applications in a growing number of areas. An area of particular interest, and the topic of this review, is gene therapy. Gene therapy refers collectively to methods aimed at influencing gene expression in living organisms through delivery of integrating or non-integrating exogenous DNA or RNA to treat or prevent diseases. The Center for Biologics Evaluation and Research (CBER), a division of the United States Federal Drug Administration (FDA) has yet to approve any human gene therapy products for sale in the U.S. However, in 2012 the European Medicines Agency (EMA) approved Glybera (alipogene tiparvovec) as the first gene therapy treatment for sale in the European Union. It is an adeno-associated virus (AAV)-mediated delivery of DNA to treat lipoprotein lipase deficiency [3].

The approval of Glybera represents the culmination of many years of research on DNA delivery with the help of viruses, including gamma-retroviruses, lentiviruses, herpesviruses adenoviruses, and the abovementioned AAV [4]. Viral vector systems are attractive because of their high transfection efficiency, the ability of some viruses (e.g., retroviruses) to integrate the transgene into the host genome [5], which is currently difficult to accomplish with non-viral methods. AAV is an especially promising vector, because it is non-pathogenic and can reliably integrate in a specific site on human chromosome 19 [6,7]. However, despite these benefits, viral vectors frequently activate the host’s immune system [5], which can reduce the effectiveness of subsequent gene delivery. For example, adenoviruses cause a very strong immune response, which activates the Toll-like receptor (TLR) independent and dependent innate immune system signaling pathways, via the detection of vector DNA [8]. Additionally, viral glycoproteins can induce the activation of the adaptive immune response, which creates immunological memory and can reduce effectiveness of future use of that viral vector [8].

**Figure 1:**
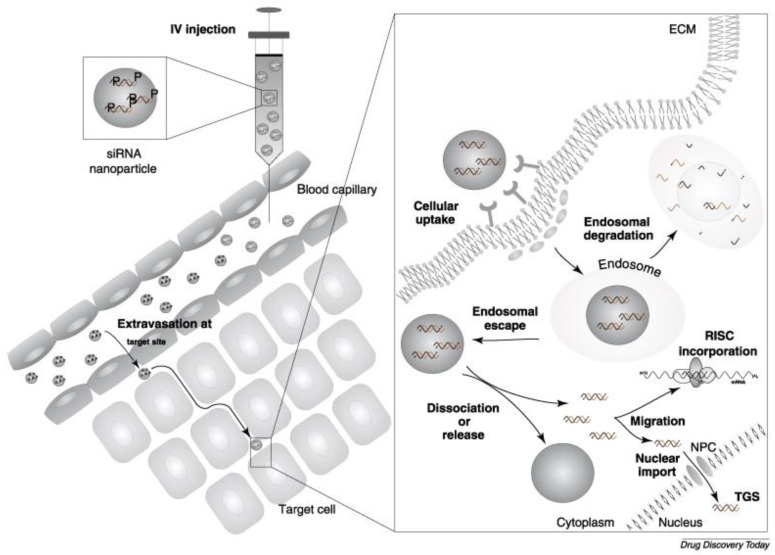
Overview of biological barriers for small interfering RNA (siRNA) therapy following intravenous (IV) injection. ECM = extracellular matrix; RISC = RNA-induced silencing complex; NPC = nuclear pore complex; TGS = transcriptional gene silencing. Reproduced with permission from [12]. Copyright Elsevier, 2008.

Delivery of DNA via non-viral vectors offers the prospect of avoiding the immune response, and can potentially also manage larger payloads. The recent developments in nanomaterials, materials science, and polymer engineering have marked a turning point in the understanding of nanoscale delivery. These advances are helping to overcome many of the disadvantages of non-viral vectors by increasing their transfection efficiency and navigating through the multiple biological barriers [9,10,11]. Aside from the need for the non-viral vectors to penetrate the cell membrane, a major challenge is to evade endosomal degradation after endocytic uptake (Figure 1) [12]. Cargo internalized via endocytosis is delivered to the early endosome to be sorted/routed to the lysosome for degradation [13], to the trans-Golgi network for processing [14], or recycled back to the plasma membrane [15].

This review summarizes recent developments related to various non-viral gene delivery systems with a basis in nanotechnology. The review is organized based on the nature of the nanomaterials: inorganic; graphene (carbon nanotubes); proteins and peptides; lipids; and other polymers.

Regardless of the vector, there are two main approaches to affecting the genetics of targeted cells: (1) Gene therapy where DNA is delivered with the aim of providing a functional copy of a defective gene in the patient, and (2) The delivery of therapeutic nucleic acids which include microRNA (miRNA), short hairpin RNA (shRNA), antisense oligonucleotides (AONS) and small interfering RNA (siRNA). In the case of miRNA, shRNA, and siRNA, the RNA species are processed via the Dicer complex, and loaded into the RNA-induced silencing complex (RISC), which then binds to messenger RNA (mRNA) molecules to either degrade them or modulate their expression [16,17,18]. By contrast, AONS are delivered as a single-stranded species, and must find their complementary mRNA sequences without the aid of an auxiliary protein (such as Argonaute in the RISC complex) [16]. This approach is typically used to target tumors, but can also be used when a genetic disorder results in elevated levels of gene expression. Examples of both strategies will be highlighted.

## 2. Inorganic Nanomaterials for Gene Delivery

When designing nanomaterials, their physical properties, such as size, shape, charge density, elasticity, and colloidal stability are important attributes that determine their suitability for a specific function [19]. Inorganic nanomaterials are highly sought after because of their ease of functionalization, unique electrical and optical properties, biocompatibility, as well as low cytotoxicity [20]. Some of the inorganic materials used as nanomaterials are gold, silver, calcium phosphate, graphene oxide, quantum dots, and magnetic nanomaterials such as iron oxides.

Gold nanomaterials have a flexible surface, which assists in their functionalization. This allows DNA to be complexed directly to gold nanoparticles (Au-NP). Son et al. [21] immobilized three functional sections of nucleic acids to the surface of an Au-NP. The goal was to create a pH-sensitive DNA-Au nanomachine, which could be used to silence *polo-like kinase 1* (*PLK1*) via siRNA. *PLK1* encodes an enzyme necessary for genomic stability and successful mitosis [21]. Thus, silencing this gene would induce apoptosis (programmed cell death) of the target cell. A pH-gradient causes a conformational change in the nucleic acid structure, which induces aggregation of the Au-NP, promoting endosomal escape, thereby releasing the siRNA. The Au-NP aggregates can also be used to cause photothermal ablation, thereby promoting a synergistic effect with the siRNA to destroy cells. Photothermal ablation is the use of laser light to generate heat locally, which then destroys cells. It was also discovered that the more compact the aggregates are, the lower the laser fluence (13 W/cm^3^ for Au-NP) needed to be, to induce photothermal ablation, thus minimizing negative impacts on surrounding cells [21]. In another study using gold nanomaterials, Peng et al. [22] used antimicrobial peptides (PEP) derived from lactoferrin to coat Au-NP. This coating was used to enhance the delivery of Au-NP to bone-marrow-derived mesenchymal stem cells (MSC). The highest transfection ability was observed when the peptide content was 2.5% of the total mass of the Au-NPs [22]. Additionally, the PEP-coating gave the Au-NP antimicrobial activity, which successfully prevented growth of *Staphylococcus aureus*, a Gram-positive bacterium, and *Escherichia coli*, a Gram-negative bacterium [22]. Trans-activator of transcription (Tat) derived from HIV-1 is a key cell-penetrating peptide [23,24]. The ability of Tat to facilitate the uptake of various molecules makes it an excellent choice to functionalize nanoparticles. Peng et al. [25] functionalized Au and silver (Ag) with Tat peptides to determine their effectiveness in penetrating epidermal stem cells (ESC). Stem cells are very difficult to penetrate with conventional non-viral vectors because of their restricted cellular uptake [26,27]. Tat peptide conjugation with Au/Ag-NP enabled the delivery of DNA to the nucleus of stem cells, with low cytotoxicity [25].

Graphene, an allotrope of carbon, is an attractive nanomaterial because of its optical, thermal, and electrical properties [28]. Graphene oxide (GO) exhibits π-π-stacking interactions (non-covalent interactions between aromatic rings), which allows for a high drug loading efficiency, as well as a controlled release [29]. Another benefit of GO, specific for gene delivery, is its ability to protect nucleotides from cleavage [30]. Kim et al. [31] designed a stimuli-responsive gene delivery vehicle, composed of polyethylene glycol-branched polyethylenimine-reduced graphene oxide (PEG-BPEI-rGO). This stimuli-responsive delivery was accomplished by using near-infrared (NIR) irradiation that, when absorbed by the GO nanoparticles, caused the temperature to increase locally, to the point where the endosome ruptured. This was termed photothermal transfection (PTT) [31]. The researchers demonstrated that the optimal transfection conditions were at 808 nm (6 W/cm^3^) for 20 min [31]. Owing to its high loading capacity, Zhi et al. [32] used GO in a nanocomplex with polyethylenimine (PEI) and poly (sodium 4-styrenesulfonates) termed PPG to deliver both microRNA-21 (mir21) and Adriamycin (ADR), an anti-cancer drug [32]. Both PPG_ADR_ and PPG_ADR+mir21_ complexes were tested in MCF-7 (human breast adenocarcinoma cells) and MCF-7/ADR, an Adriamycin-resistant breast cancer cell line. PPG_ADR+mir21_ reduced the cell survival rate from 35% to 28% in MCF-7 cells, and from 52% to 30% in MCF-7/ADR when compared to PPG_ADR_. This effectively demonstrated that PPG_ADR+mir21_ was able to overcome tumor multidrug resistance.

**Figure 2:**
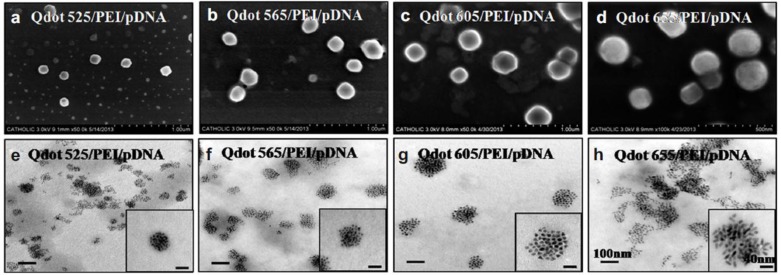
Scanning electron (SE) micrographs (**a**–**d**) and transmission electron (TE) micrographs (**e**–**h**) of multiple quantum dot (QD) nanoparticles complexed with plasmid (p) DNA. The sizes of the QDs is indicated in the images. The numbers 525, 565, 605, 655 refer to the wavelength (in nm) at which the emission spectrum of the respective QDs peaks. Scale bars in the SEM images indicate 100 μm, and in the TEM images 100 nm. Reproduced with permission from [36]. Copyright Elsevier, 2014.

Quantum dots (QD) are semiconductor-based, monodisperse, nanometer-sized crystals (a.k.a. nanocrystals) [33]. Quantum dots can be synthesized with different methods, including colloidal [33] or plasma synthesis mechanisms [34]. Quantum dots (QD) are similar to graphene in that they are attractive inorganic materials because of their optical and electrical properties [35]. The size of quantum dots was shown to be directly related to their uptake and gene delivery efficiency [35]. Yang et al. [36] fabricated multiple QD (MQD) bundles by coating a single dot (SD) with PEI (Figure 2). These MQDs were able to bind a plasmid DNA molecule (pDNA) encoding enhanced green fluorescent protein (pEGFP), and effectively delivered it into human mesenchymal stem cells (hMSCs). When evaluating the transfection efficiency of different sizes of QDs (QD525, QD565, or QD605, and QD655, ranging from 5 to 20 nm in diameter), the largest (QD655, coated with PEI/pDNA) resulted in the highest transfection efficiency, evident by a 60% greater fluorescence intensity compared to that observed when using QD525 [36]. Quantum dot gene delivery provides another avenue to target difficult-to-penetrate stem cells [26].

Magnetic nanoparticles (MNP) such as iron oxides are unique in their ability to provide magnetic resonance imaging (MRI) as well as to carry a therapeutic payload [37]. While there is a wide range of potential MNPs, γ-Fe_2_O_4_ is the most promising, because it has the lowest toxicity [38]. Kami et al. [39] used MNP (γ-FeO) conjugated with PEI to design magnetoplexes to transfect mouse embryonic fibroblasts with a plasmid encoding GFP. These researchers demonstrated that there was a 6–8 fold higher GFP expression in the PEI-MNP with a magnetic field, versus PEI or PEI-MNP without a magnetic field. Additionally, the GFP expression was sustained for up to seven days in the PEI-MNP with a magnetic field [39]. The magnetic properties conferred by the iron oxide not only allowed the tracking of the magnetoplexes in vivo, but also enabled cell separation [39]. Superparamagnetism occurs when a nano-sized particle undergoes the transition to the paramagnetic state below the Curie temperature. Materials in the paramagnetic state have induced magnetization in the presence of a magnetic field, and no magnetization in the absence of a magnetic field. Superparamagnetic iron oxide nanoparticles (SPIONs) are becoming an increasing popular drug delivery choice because a magnet can be applied to target SPIONs to a specific location, and once the magnet is removed, magnetic interactions between the particles cease, preventing large colloidal aggregations of SPIONs [40]. SPIONs coated with PEI have previously been used to deliver genes to cancer cells, yet the functionalization of these particles with PEI resulted in high toxicity [41]. Li et al. [42] designed a bioreducible cationic polymer-coated SPION for siRNA delivery and MRI. The result was SSPEI (disulfide-polyethylenimine)—SPION, a multifunctional theranostic platform. SSPEI-SPION was able to effectively deliver human telomerase reverse transcriptase (hTERT) siRNA with low cytotoxicity, which represented a major contrast with PEI-SPION [42]. Voronina et al. [43] used SPION and PEI bound by biotin-streptavidin to create a multifunctional nanocomplex consisting of miRNA-PEI-SPION, and which resulted in highly efficient transfection. The cytotoxic effect of PEI was offset by an increase in iron loading present in the miRNA-PEI-SPION complex, due to a reduction of PEI necessary for miRNA delivery [43].

## 3. Carbon Nanotubes for Gene Delivery

First described in 1991 [44], carbon nanotubes (CNTs), formed from one or more sheets of graphene shaped in a cylindrical structure, have unique chemical and physical properties. Iijima [44,45] initially discovered multiwalled carbon nanotubes (MWNTs), which have a diameter of 4–30 nm, with two or more graphene cylinders centrically arranged. Two years later Iijima and Ichihashi [45,46] reported on single walled carbon nanotubes (SWNTs), which have a diameter of 0.4 to 3 nm, and which are composed of a single graphene sheet. While their small size and chemical inertness are attractive properties for delivery of DNA, their hydrophobic nature makes them poorly soluble in aqueous solutions, which places limitations on their application in biological systems. To increase their solubility and dispersion, CNTs can be functionalized through either covalent or non-covalent functionalization [47,48].

The two most common types of covalent functionalization reactions are oxidations and cyclo-additions. While covalent functionalization can improve the biocompatibility and solubility of pristine (chemically unmodified) carbon nanotubes, many of their intrinsic physical properties are either destroyed or weakened [47]. Alternatively, non-covalent functionalization can be carried out by coating the carbon nanotube with amphiphilic molecules, such as sodium dodecyl sulfate [SDS] [49] or proteins [50], which preserves the chemical structure, allowing the CNTs to maintain their unique physical properties [51,52,53,54].

Covalent functionalization of CNTs potentially limits the DNA loading capacity of a CNT-based gene delivery system. Consequently, Wang et al. [55] delivered siRNA (as opposed to delivering a larger gene payload) against hTERT, using a covalently functionalized SWNT-PEI/NGR/siRNA system. NGR is a tumor-targeting peptide (Cys-Asn-Gly-Arg-Cys-) used to increase the transfection efficiency of the vector system into human prostate cancer cell line PC-3 [55]. Despite the covalent functionalization, SWNTs still possess the inherent ability to absorb radiation in the near-infrared region [56]. Thus, in addition to utilizing the delivery of siRNA, Wang et al. [55] utilized NIR irradiation with a wavelength of 808 nm (based on a previous study [57]) to induce apoptosis in the target cells. A therapy using a combination of phototherapy and siRNA delivery could offer an alternative treatment to eliminate tumors.

Polyethylenimine is a very popular transfection agent (see Section 6) that despite its severe toxicity [58] is often utilized for various functionalization techniques [59]. Behnam et al. [60] demonstrated that non-covalent attachment of PEI to SWNTs could enhance the transfection efficiency compared to the use of PEI by itself. There was a direct correlation between the molecular weight of PEI used, and transfection efficiency, while the correlation was inverse between molecular weight and cytotoxicity. Chemically modified PEI was shown to have reduced toxicity. Kong et al. [61] demonstrated that PEI-cholesterol (Chol) could be non-covalently bound to SWNTs, enhance gene delivery, while reducing the toxicity relative to unmodified PEI. This novel PEI-Chol-SWNT (PCS) system also had photothermal properties, which were exploited to promote release of DNA upon exposure to NIR. This photothermal irradiation shows increased transfection with pEGFP (plasmid DNA encoding enhanced green fluorescent protein) and apoptosis in human cervix adenocarcinoma cells (HeLa) cells, while promoting anti-tumor activity in MCF-7 cells versus using the PCS complex with NIR irradiation [61]. Succinated PEI (PEI-SA) was utilized by Siu et al. [62] to non-covalently functionalize SWNTs to topically deliver siRNA to treat melanoma. The group demonstrated that PEI-SA/CNT/siRNA could retard tumor growth in C57bl/6 mice, as well as silence the *Braf* gene, which is a proto-oncogene encoding the protein B-Raf, in B16-F10 melanoma cells.

**Figure 3:**
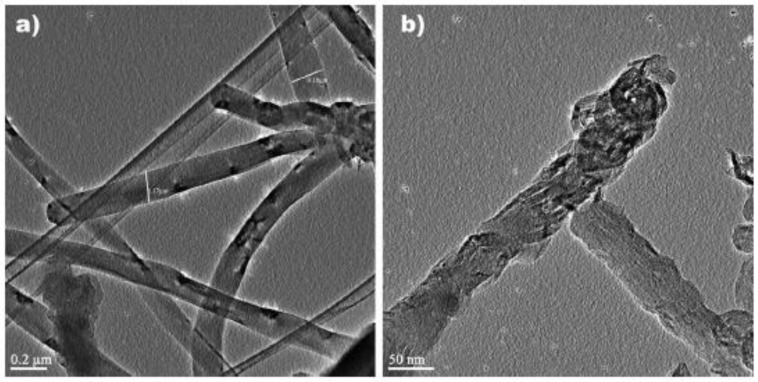
(**a**) Transmission electron micrographs of unmodified carbon nanotubes (CNTs) and (**b**) functionalized CNTs with activated carboxyl moieties. Scale bar equals 0.2 μm in (a); 50 nm in (b). Figures reproduced with permission from [64]. Copyright John Wiley & Sons, 2013.

MWNTs are generally considered less attractive than SWNTs because of their larger diameter. However, the large diameter is beneficial for the delivery of larger payloads of DNA. Additionally, the toxicity is not based solely on the size of the nanotube, but is also determined by the method of functionalization [47]. Liu et al. [63] covalently functionalized MWNTs with chitosan-folic acid nanoparticles (CS-FA NPs) to deliver plasmid DNA. The group demonstrated that shorter MWNTs have a higher transfection efficiency, but as a consequence, also have a higher cytotoxicity. Yet, when functionalized with CS-FA NPs, the transfection efficiency was increased, while the cytotoxicity decreased [63]. Geyik et al. [64] delivered a linearized plasmid via covalently functionalized carboxylated MWNTs (fCNT) (Figure 3a,b). Amino groups were introduced into the plasmid DNA, resulting in amino-modified plasmid DNA (mpDNA). The activated fCNTs were complexed with mpDNA to form an active bioconjugate that could be used to transform *E. coli* with greater transformation efficiency, and offer a potential alternative to electroporation or heat shock-induced transformation [64]. Jain et al. [65] designed a different bioconjugate to deliver a plasmid to MCF-7 or HeLa cells. This bioconjugate consisted of estradiol-functionalized MWNTs, polyethylene glycol (PEG) as a spacer and then stabilized with poly-l-lysine. Estradiol acted as the targeting ligand, and the transfection efficiency was proportional to the number of estrogen receptors expressed on the target cell. MCF-7 cells have a higher expression of estrogen receptors, leading to higher transfection efficiency than HeLa cells [65]. This demonstrated how the functionalization of the CNTs can determine both transfection efficiency and targeting of the bioconjugate.

## 4. Proteins and Peptide Nanomaterials for Gene Delivery

Proteins are attractive as gene delivery vectors, because of their biocompatibility, biodegradability and, typically, minimal toxicity [66]. Their amphiphilic nature allows them to interact with both the solvent and the specific cargo (DNA, RNA or therapeutic agent) of choice. While there are many different proteins utilized for gene delivery, gelatin is one of the most commonly used proteins [67]. Gelatin is produced by the hydrolysis of collagen, and is composed of peptides of varying length. Depending on whether the gelatin was isolated from tissue with acid or base, it is referred to as type A or type B. The main difference between the two types is the iso-electric point [68]. Various attributes of gelatin that make it attractive are the ease of modification and low cost of production. Lee et al. [69] demonstrated the ease of gelatin modification by designing a thiolated gelatin (tGel) nanoparticle that could be chemically cross-linked to a polymerized siRNA (poly-siRNA). The siRNA was polymerized with thiol groups on the 5′-end of the sense and anti-sense strands, to allow interactions between the tGel and siRNA, which resulted in a poly-siRNA-thiolated gelatin (psi-tGel) [69]. The researchers effectively utilized the psi(RFP)-tGel NPs to induce gene silencing in RFP/B16F10 melanoma cells, which was demonstrated by an 80% reduction in *RFP* mRNA expression relative to the control [69]. Moran et al. [70] utilized gelatin B and protamine sulfate to deliver DNA. The use of gelatin B was an innovative choice, because it has an isoelectric point (pI) of 4.8–5.2, which makes it negatively charged at physiological pH and allows interactions with oppositely charged molecules [71]. However, when gelatin B encounters an endosome, gelatin B becomes positively charged, releasing the therapeutic agent associated with it into the cell. Protamine sulfate (PS) is a highly positively charged molecule that can bind DNA, offering a mechanism to trap the DNA inside the gelatin B–PS complex for efficient gene delivery. Moran et al. [70] demonstrated that DNA release is dependent upon the initial DNA concentration and the gel strength of the gelatin. The maximum amount of DNA released was reported to be between 12 and 17 μg·mL^−1^ depending on the exact gelatin configuration.

Albumin, although not considered a gene delivery vector per se, is often utilized to assist other molecules in the delivery of their gene cargo and is usually obtained from either bovine serum albumin (BSA) or human serum albumin (HSA) [66]. Albumin is a major blood plasma protein that is easily modified because of the many reactive groups on the protein surface. Additionally, it is an attractive cellular carrier for cancer treatment, because albumin accumulates in tumors [72,73]. Karimi et al. [74] demonstrated the use of a core-shell structure for potential co-delivery of genetic material and/or drugs. The core was composed of albumin (Alb) and the shell was composed of chitosan (CS), which can interact with DNA, creating a novel Alb-CS-DNA complex. This complex was used to deliver a plasmid encoding short hairpin RNA (shRNA) against GL3 luciferase, into HeLa cells. Cellular uptake was monitored by flow cytometry originating from FITC-labeled Alb-CS-DNA NP. Karimi et al. [74] concluded that 85% of the HeLa cells contained the Alb-CS-DNA NP, and that there was minimal toxicity. Additionally, they demonstrated that Alb imparts biocompatibility to the Alb-CS-DNA NP when viability levels were compared to NPs consisting of either just Alb or CS [74]. Han et al. [75] modified the surface of BSA with ethylenediamine to create a cationic BSA (CBSA). Simply mixing CBSA with siRNA resulted in a CBSA/siRNA-NP via electrostatic interactions. The CBSA not only protected the siRNA from RNA degradation, but was also able to efficiently deliver the siRNA into B16 lung metastatic cells [75].

**Figure 4:**
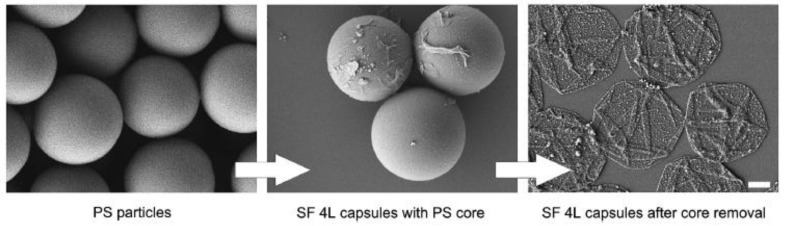
Representative scanning electron micrographs showing surface morphology of 4-μm polystyrene (PS) particles, after four layers (4L) of silk fibroin (SF) coating, and the dried microcapsules after core removal. Scale bar: 1 μm. Figures reproduced with permission from [78]. Copyright Elsevier, 2014.

Silk is a natural protein that is spun into fibers by various arthropods, mostly during the metamorphosis stage of their development. Silk protein can vary widely in composition, yet the best studied silk comes from the silkworm *Bombyx mori* and the spiders *Nephilia clavipes* and *Araneus diadematus*. The highly repetitive nature of amino acids in silk proteins causes silk to exhibit mechanical properties that can be exploited in tissue engineering [76]. Recent advancements in materials science have shown that ultra-thin silk fibroin (SF) can potentially be used as a drug/gene delivery system [77]. Li et al. [78] used layer-by-layer assembly onto a polystyrene template (Figure 4) to design an SF vector to deliver pDNA to transfect NIH/3T3 fibroblasts. Also, Zeta-potential measurements determined the optimal number of SF coatings necessary to maximize the pDNA adsorption onto the particles. The group demonstrated that plasmid DNA loaded onto SF microcapsules could efficiently transfect NIH/3T3 fibroblasts (both 1-μm and 4-μm microcapsules) and that the DNA loading method (either pre- or post-SF deposition) influenced the transfection efficiency [78].

Zein is a storage protein present in the seeds of maize (*Zea mays* L.). It is a member of a class of plant storage proteins called prolamines, which contain a high percentage of the amino acid proline. One of the defining characteristics of zein is its insolubility in water due to the high concentration of amino acids with hydrophobic side chains [79]. This hydrophobic nature has been utilized for the sustained delivery of DNA [80]. Zein is considered a “generally-regarded-as-safe” (GRAS) polymer, and has been approved by the FDA for human use. Karthikeyan et al. [81], extending the work of Regier et al. [80], fabricated zein nanofibers to determine whether they could be used for the sustained delivery of siRNA into skin fibroblast cells for gene silencing. The group reported that the zein-nanofiber was able to controllably release siRNA for 72 h. Additionally, only a total of 34% (w/w) of initially entrapped siRNA was detected in the solution after 72 h. The fact that there was still a substantial amount of siRNA entrapped in the zein nanofibers suggested the potential for gene silencing for periods beyond 72 h [81].

Elastin is a protein in connective tissue that provides elasticity. Both α-elastin and elastin-like polypeptides (ELPs) have been utilized in drug/gene delivery applications. ELPs are artificial peptides with the protein sequence (Val-Pro-Gly-X-Gly)*_n_* (with X capable of being any amino acid, and *n* = the number of repeat units) [66]. These ELPs show thermal phase transitions, either above or below their transition temperature (T_t_). ELPs are soluble below their T_t_, while they form a viscous fluid called coacervate above the T_t_ [82]. The fourth amino acid choice in the repeat unit (X) allows the ELPs to have a tunable T_t_ [82]. Dash et al. [83] designed a dual ELP-based delivery system to deliver two different gene payloads. This ELP-injectable system was composed of an ELP gel scaffold and ELP hollow spheres that had been used for gene delivery previously [84]. This dual system allowed them to deliver two different plasmids, one encoding endothethial nitric oxide synthase (eNOS) in the ELP hollow spheres, and a second one encoding interleukin-10 (IL-10) in the ELP gel scaffold, to human umbilical vein endothelial cells (HUVEC) cells, to modulate angiogenesis and inflammation to treat critical limb ischemia [83]. The DNA release rate corresponded to the degradation rate of the polymer, with the scaffold degrading first. Overall, this study [83] demonstrated controlled release, with this particular treatment leading to a reduction in inflammation and an increase in blood vessel density.

Poly(aspartic acid) (PAsp) nanoparticles have recently emerged as a potential short peptide delivery system [85]. Dou et al. [86] designed both linear and star-shaped PAsp vectors, functionalized with poly-(2-(dimethylamino) ethyl methacrylate) (PDMAEMA). PDMAEMA by itself enables high transfection efficiency, but is highly toxic to cells [87]. PAsp-PDMAEMA polymer was used to deliver plasmid DNA encoding Renilla luciferase (pRLCMV) into HepG2 and COS7 cells, which demonstrated high transfection efficiency, particularly among the star shaped PAsp vectors, with reduced toxicity [82]. Unzueta et al. [88] utilized poly-arginines (R9), poly-histidines (H6), and GFP (used as a scaffold) [R9-GFP-H6] as a DNA delivery vector. In the presence of DNA and at a slightly acidic pH, the hexa-histidine tails undergo rearrangement and assemble in a mechanism similar to that observed for virus coat proteins [88]. This rearrangement yielded both nanosphere and nanotube structures. R9-GFP-H6-DNA was demonstrated to be able to transfect HeLa cells and protect the DNA from degradation by deoxyribonuclease I (DNase I) [88].

## 5. Lipid-based Nanomaterials for Gene Delivery

**Figure 5:**
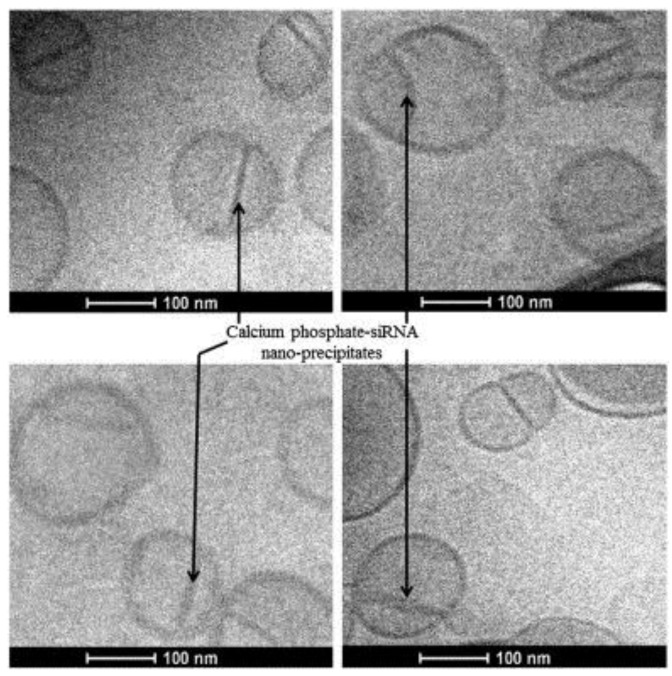
Transmission electron micrographs of cyclic arginine-glycine-aspartic acid (cRGD)—CPE liposomes (2%). CPE refers to calcium phosphate encapsulated siRNA. Arrows point to siRNA-calcium phosphate nano-precipitates inside the liposomes. Scale bar: 100 nm. Figure reproduced with permission from [92]. Copyright Elsevier, 2014.

Lipids are a class of biological molecules defined by their hydrophobic and/or amphiphilic nature. This includes fats, waxes, oils, cholesterols, phospholipids, glycerides, and the fat-soluble vitamins A, D, E, and K. The amphiphilic nature of lipids and their ability to form vesicles and membranes make lipid-based delivery systems attractive. Felgner et al. [89] were the first to demonstrate lipid-based gene delivery, by utilizing a liposome containing the cationic lipid (±)-*N*,*N*,*N*-trimethyl-2,3-*bis*(*z*-octadec-9-enyloxy)-1-propanaminium chloride (DOTMA) to deliver plasmid DNA into eukaryotic cell lines. While anionic, cationic, or neutral lipids can be employed to deliver DNA into cells, cationic lipids are preferred because of their ability to adsorb efficiently onto the anionic cellular membrane [90]. Thus, cationic lipids/DNA (lipoplexes) offer greater transfection efficiency [91]. When designing the lipoplexes, there is usually some combination of a cationic lipid and neutral lipid (or helper lipid). These neutral lipids are helpful in the formation of a lipid bilayer. Some of the common helper lipids are cholesterol and dioleoylphosphatidyl ethanolamine (DOPE) [91]. Khatri et al. [92] designed a liposome composed of dipalmitoylphosphatidyl choline (DPPC), DOPE, cholesterol and a PEGylated lipid (DSPE-mPEG) to deliver siRNA (CPE liposome) complexed with calcium phosphate. The calcium cations were able to complex with siRNA. Additionally, the liposome was able to protect the siRNA from degradation. The liposome was further modified with cyclic arginine-glycine-aspartic acid (cRGD) (Figure 5) to target the liposome complex to A549 lung cancer cells [93]. The cRGD-CPE liposomes were more effective at inhibiting *RRM1* (76% reduction in *RRM1* expression), than the CPE-liposomes (70% reduction) and naked siRNA (16% reduction) [92]. Kullberg et al. [94] designed a two-component delivery system utilizing neutral liposomes to deliver plasmid DNA encoding a luciferase reporter. The plasmid DNA was first condensed with PEGylated cationic poly-lysine to form PL/DNA. Next, the liposome was conjugated with Listeriolysin O (LLO), a pore-forming protein, to target the liposome to human epidermal growth factor receptor—2 (Her-2) in breast cancer cells [94]. Isogenic cell lines MCF-7 and MCF-7/Her18 cells were selected, with MCF-7/Her18 overexpressing the Her-2 cell surface receptor [95]. The group demonstrated that PL/DNA co-localizes with LLO/Liposomes 68% of the time in the MCF-7/Her18 cells, while only 0.7% of the time in the MCF-7 cells, indicating the ability of LLO to act as a targeting ligand for the Her-2 receptor. Finally, the group observed a 268-fold greater luciferase expression in the MCF-7/Her18 cells versus MCF-7 cells, demonstrating efficiency and specificity of the two-component system [94].

Small lipid like molecules known as lipidoids have recently been investigated for their use as RNA interference gene delivery vectors [96]. In contrast to cationic lipids, lipidoids have characteristic cationic behavior due to their amine backbone [97]. Lipidoids are synthesized using a Michael addition reaction, which adds an amine to either an acrylate or acrylamide group [97]. Additionally, lipidoids can have between one and seven tails, depending on the amine used in the addition reaction, which contrasts with natural lipids having only two tails [96,97]. Knapp et al. [98] demonstrated that lipidoid nanoparticles [LNPs] could be utilized to deliver siRNA to mantle cell lymphoma cells. Lipidoid 306O_13_ was selected based on a study by Whitehead et al. [96], demonstrating its efficacy in many different cell types. This LNP was used to deliver siRNA against the anti-apoptotic protein Mcl-1. The *Mcl-1* mRNA expression levels were reduced by 80% by day 3, and maintained at least 60% gene silencing for over a week [98]. It was also demonstrated that when *Mcl-1* was effectively silenced, there was an increase in the number of cells undergoing apoptosis. During the first two days, 5–15% of the cells underwent apoptosis, which increased to 30% by day 3. The cells treated with the control LNP (siRNA absent) did not show a decrease in *Mcl-1* expression, nor an increase in apoptosis rates [98]. In another study, Moon et al. [99] demonstrated that LNPs can be used to deliver siRNA into hepatocytes to inhibit Hepatitis C (HCV) viral replication. Lipidoid 98N_12_-5 was utilized as the siRNA delivery vector based on previous research illustrating its efficiency in RNA interference [100]. An siRNA against protein kinase C-related kinase (PRK2) was selected to determine if there was a reduction in RNA-dependent RNA polymerase activity needed for HCV replication [99]. After the group tested 11 different siRNA molecules against different regions of the *PRK2* mRNA, the maximum reduction in HCV RNA levels achieved was 56% [99]. They demonstrated that the LNP-siPRK2-1 was able to reduce PRK2 levels in mice hepatocytes by 30% over control mice hepatocytes injected with naked siRNA. Additionally, it was demonstrated that LNP-siPRK2-1 was able to reduce PRK2 levels by 75% in cultured human liver (Huh7) cells [99].

Another class of lipid molecules used for gene delivery is the gemini surfactants. Gemini surfactants have a basic structure consisting of at least two hydrophobic tails and two polar head groups linked by a spacer molecule [101]. With so many variations existing for the three components of gemini surfactants, a wide range of compounds can be synthesized. This has led to four major classes of gemini surfactants: (1) m-s-m (*N*,*N*-bis(dimethyl alkyl)-α,ω-alkanediammonium), (2) carbohydrate-based, (3) peptide-substituted, and (4) disulfide-containing gemini surfactants [102]. Gemini surfactants have become an attractive gene delivery system because of their low synthesis cost and ability to bind DNA. Alqawlaq et al. [103] designed a gemini surfactant phospholipid nanoparticle (GL-NP) complexed with plasmid DNA to target rat retina ganglion (RGC-5) cells to treat glaucoma. The addition of a helper lipid enhanced the gene delivery vector, creating PGLNPs [103]. The effects of topical application were investigated, as well as intravitreal injection (i.e*.*, into the eye) to determine the localization of the PGLNPs by using Cy5-labeled plasmid DNA. The group demonstrated that following intravitreal injection the PGLNPs were localized within the nerve fiber layer (NFL), ganglion cell layer (GCL), and inner plexiform layer (IPL) of the retina [103]. Yet, with topical application the PGLNPs were localized near the iris, limbus, and conjunctiva of the eye [103]. The differences in localization based on application method could be utilized to target different glaucoma-related tissues.

## 6. Polymer-Based Nanomaterials for Gene Delivery

Both natural and synthetic polymers display variation in structure (e.g., linear or branched), and molecular weight, that together with the chemical properties of the repeat unit(s), affect their physico-chemical properties. Due to the great diversity in polymers, polymer-based nanomaterials have drawn interest as potential delivery vectors. Electrostatic interactions between DNA and polymer allow nanocomplexes to be formed with DNA, protecting the DNA from degradation. There are, however, two major hurdles that must be overcome for polymer-based nanomaterials to be used as efficient gene delivery systems. First, when these polymer nanomaterials are internalized into the cell (e.g., via the endocytic pathway), there must be a mechanism for the nanomaterial to escape the endosome before it is degraded via fusion with a lysosome. Secondly, the fate of the nanomaterials under physiological conditions must be determined, especially if there are undesired interactions with proteins or other serum components that can result in aggregation.

The proton sponge mechanism has been hypothesized to explain how polymer nanomaterials escape the endosome [104]. For example, the aforementioned polyethyleneimine (PEI) has a high pH buffering capacity, which allows it to take up protons that are pumped into the endosome. This results in passive diffusion of chloride ions, which causes water influx. This increased osmotic swelling causes the endosome to rupture, causing the release of the polymer nanomaterial [104].

Incorporation of biodegradable segments into a polymer structure not only increases the biodegradability of the polymer, but also facilitates the unpacking of genes in the core of a nanocomplex, increasing transfection efficiency. Derivatization with polyethyleneglycol (PEG; PEGylation) can be used to shield a nanocomplex from interactions in the extracellular environment, preventing aggregation and clearance by the reticuloendothelial system (RES) [105,106].

### 6.1. Polyethyleneglycol (PEG)

Chen et al. [107] synthesized cationic polylactides (CPLAs), a natural degradable biomaterial, to serve as a delivery vector for siRNA into prostate cancer cells. While CPLAs resulted in higher transfection efficiency compared to a commercial transfection agent, FuGENE 6, CPLAs had reduced transfection efficiencies in the presence of serum. Albumin, present in high concentration in serum, associated with CPLAs, promoting aggregation and subsequent clearance via the RES system [105]. Chen et al. [108] synthesized a PEG block CPLA copolymer (PEG-*b*-CPLAs), to assess both transfection efficiency of the system and the stability of the system in the presence of serum. This group compared two copolymers, PEG-*b*-CPLAs-20 and PEG-*b*-CPLAs-50 (the number indicates amine molarity percentage) in terms of transfection efficiency and degradation rate [108]. The degradation rate was higher when the amine concentration in the copolymer was greater. Furthermore, PEG-*b*-CPLAs-50 interacted strongly with pDNA, with a polymer/DNA ratio of 256:1, whereas the 512:1 ratio of PEG-*b*-CPLAs-20:pDNA indicated a less strong interaction. Compared to FuGENE 6, PEG-*b*-CPLAs-50 performed similarly at low polymer/DNA mass ratios, but performed much better at a higher polymer/DNA mass ratio. The stability of PEG-*b*-CPLAs-50 in the presence of different concentrations of fetal bovine albumin (FBS) was also established. FuGENE 6 and CPLA-50 both had significant reductions in transfection efficiency with increasing FBS concentrations, whereas PEG-*b*-CPLAs-50 only displayed a minimal decrease. These observations further support the ability of PEG to shield CPLAs from the extracellular environment, preventing protein aggregation and subsequent clearance via RES [108].

### 6.2. Polyethyleneimine (PEI)

Polyethyleneimine is utilized as a gene delivery vector because of its ability to condense DNA into polyplexes, but also because of effectiveness at escaping endosomes via the proton sponge hypothesis, mentioned above. Unmodified PEI is, however, cytotoxic and has a low transfection rate due to its high positive charge [58,109]. To reduce the cytotoxicity of PEI, He et al. [110] coated PEI/DNA complexes with a disulfide-modified hyaluronic acid (HA-SS-COOH) to assess transfection efficiency. Hyaluronic acid (HA) also has targeted uptake via HA receptor-mediated endocytosis [111]. He et al. [110] demonstrated greater degree of shielding and higher transfection efficiency of their DNA-PEI-HA-SS-COOH (DPS) vector up to about 14-fold, over both DNA-PEI-HA (DPH) and DNA-PEI (DP) in the presence of different concentrations of fetal bovine serum. This group also demonstrated how the presence of the HA receptor influences transfection efficiency, with DPS having a higher transfection efficiency in HepG2 cells [overexpressing the HA receptor] than DP, whereas in NIH3T3 cells [HA receptor deficient] DP had a similar transfection efficiency as DPS [110]. The effect of HA receptors on transfection was further confirmed via a HA competition assay, in which HepG2, B16F10 [HA receptor positive] and NIH3T3 cells were pretreated with free HA. In NIH3T3 cells the DPS transfection efficiency was not affected, but DPS transfection efficiency decreased in both HepG2 and B16F10 cells. This demonstrated how the DPS vector has greater cellular uptake in the presence of HA receptors, and how the shielding ability resulted in lower cytotoxicity and transfection [110].

Chen et al. [112] developed a pH-responsive acetylated cyclodextrin (Ac-aCD) to deliver antisense oligonucleotides (ASONs) targeted to Bcl-xL (an anti-apoptotic protein) in human lung adenocarcinoma cells. Biomaterials for gene delivery that can undergo a pH-sensitive change are highly sought after because of their ability to release cargo into the endosome, preventing degradation following transport to lysosomes. From a therapeutic perspective, a pH-sensitive biomaterial is preferred, because of the low pH in many tumors, inflammatory/infectious sites and the endosome compartment, thus helping to guarantee cargo release at the specific target site [113]. Low-M_W_ PEI1800 was hybridized to the pH-responsive cyclodextrin (Ac-aCD) to help promote endosomal escape [112]. The pH-responsiveness of the Ac-aCD nanoparticle was evident from the heightened release rate of Cy3-labeled ASON at a pH of 5 vs. 7.4. The As-aCD nanoparticle was also able to effectively inhibit cell growth. Cell inhibition responded in a dose- and time-dependent manner, with a maximum growth inhibition of 41% at 200 pmoL/mL ASON/Bcl-xL at 24 h, increasing to 80% at 72 h [112]. Finally, the group demonstrated that their Ac-aCD vector had a higher transfection and lower cytotoxicity than Lipofectamine 2000 and poly(lactic-*co*-glycolic acid) (PLGA) based nanoparticles.

### 6.3. Natural Polymer-Based Nanomaterials

**Figure 6:**
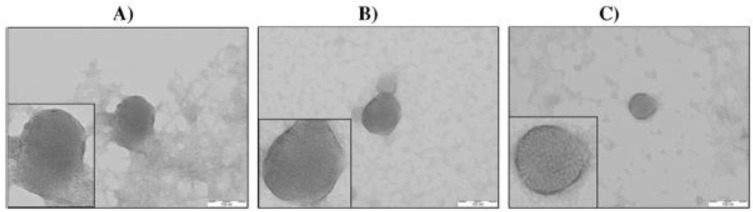
Morphology of cyclodextrin.siRNA structures visualized by transmission electron microscopy. (**A**) Cationic cyclodextrin (CCD) nanoparticle, (**B**) CD-M nanoparticle (M = methyl), (**C**) CD-F nanoparticle (F = folate). See text for full compositional details. All main structures shown at 200,000× magnification, with the scale bar indicating 100 nm. Insets show enlarged images at greater resolution. Figures reproduced with permission from [117]. Copyright Elsevier, 2016.

Cyclodextrins (CD) are cyclic polymers made up of α-1-4-d-glucose or amylose derived from the enzymatic conversion of starch [114]. Being natural polymers, CD have low immunogenicity and have been shown to interact with nucleic acids, making them an attractive choice as a gene delivery vector [115]. Godinho et al. [116] demonstrated that PEGylated cyclodextrins with increasing molecular weight PEG can be used to reduce aggregation and elimination via the RES system. However, in the same study no significant difference in siRNA delivery was observed between PEGylated-CD.siRNA and CD.siRNA [116]. Evans et al. [117] designed a CD.siRNA-DSPE-PEG_5000_-folate nanoparticle to determine the gene silencing ability in prostate cancer cell lines. Prostate-specific membrane antigen (PSMA) is upregulated in prostate cancer, and its expression increases as the cancer metastasizes. Folate was used as the targeting ligand because PSMA has been shown to enhance uptake of folate-containing particles [118]. Incorporation of DSPE-PEG5000 was utilized to reduce the overall cationic charge of the particle to limit clearance by RES system. Three different formulations of cyclodextrin complexed with siRNA were used (Figure 6): CCD (cationic amphiphilic cyclodextrin /siRNA), CD-M (Cyclodextrin/siRNA-DSPE-PEG_5000_-methyl) and CD-F (Cyclodextrin/siRNA-DSPE-PEG_5000_-folate), and their gene delivery and targeting uptake potentials were assessed in PSMA-positive and PSMA-negative cells [117]. The CD-F formulation resulted in increased targeted uptake and gene delivery potential in PSMA-positive cells (VCaP and LNCaP) compared to CD-M. Yet, when cells were pretreated with excess folate, CD-F resulted in reduced cellular uptake, while no significant change in uptake in untreated vs. folate-pretreated cells was observed for CD-M, indicating that the free folate competed with CD-F for targeted uptake [117]. Overall it was demonstrated how folate could be used both as a targeting ligand and as a method to increase cellular uptake.

Poly-l-lysine (PLL) is a peptide-based polymer, and was one of the first polymers utilized as a non-viral gene delivery vector. Its peptide structure allows ease of biodegradability and complexes with plasmid DNA to transfect cells. Compared to PEI, however, it has lower transfection efficiency resulting from endosomal capture [119]. Zeng et al. [120] designed two polyethyleneglycol (PEG)-based polymers to deliver siRNA and arsenic trioxide (ATO) to treat pancreatic cancer. PEG-PLL was designed to complex with siRNA and down-regulate mutant *Kras* gene expression in pancreatic cancer, while PEG-Poly-dl-lactide (PDLLA) was used to encapsulate and deliver ATO into pancreatic cells to induce apoptosis. BXPC-3 cell lines with the wild-type *Kras* allele, and PANC-1 cell lines with a mutant *Kras* allele were utilized to evaluate the ability to silence the mutant, but not the wild-type *Kras* allele [120]. The PEG-PLL/siKras system was able to effectively silence the mutant allele, verified at both the mRNA level (a 61% reduction in expression) and the protein level (57% reduction in expression) in PANC-1 cells [120]. The PEG-PLL/siKras system did not have any silencing effect in the BXPC-3 cells lines, demonstrating the ability to selectively target a specific allele. Administering just siKras or arsenic (As)-NPs only induced apoptosis in 13% and 36% of cells, respectively. However, when co-administered, a synergistic effect was observed with apoptosis occurring in 41% of cells. The overall cancer cell viability when PEG-PLL/siKras and PEG-PDLLA/As were co-administered decreased as the concentration of arsenic increased, resulting in a cell viability of 37% and 66% at 20 vs. 2.5 μmoL/L arsenic, respectively [120]. The results from this study clearly demonstrated the synergistic effect of combining siRNA therapeutics with an anticancer drug to treat cancer.

Lignin is a complex plant cell wall polymer formed via radical-mediated coupling of different hydroxycinnamyl alcohols and related compounds, referred to as monolignols [121]. Since lignin can be formed from different monolignols, there is considerable variation in lignin structure across plant species and among tissues and developmental stages within a given species. Caicedo et al. [122] developed a method to synthesize lignin-based nanotubes (LNTs) and nanowires using an alumina membrane as a sacrificial template. After showing the ability of DNA to adsorb onto LNTs, Ten et al. [123] demonstrated the potential of LNTs to be used as a gene delivery vector. The morphology and properties of the LNTs were dependent on the plant species from which the lignin originated as well as the lignin isolation procedure [123]. For example, LNTs synthesized with lignin that had been isolated from loblolly pine (*Pinus taeda* L) wood with thioglycolic acid localized to the cytosol of HeLa cells, whereas LNTs synthesized from pine wood lignin isolated using NaOH penetrated the nuclei of the HeLa cells. Western analysis showed that the pDNA encoding GFP that was carried by the LNTs was expressed. The combined results suggest that various properties of LNTs can be fine-tuned by selecting the source of the lignin and synthesis conditions [122,123].

## 7. Future Prospects

As is evident from this review, many new and innovative approaches to therapeutic gene delivery have emerged in recent years. Nanomaterials appear especially well suited as delivery vectors for small RNA molecules. Given that the vast majority of studies with nanomaterials have been conducted in cell culture systems, it is also clear that additional tests with whole organisms will be necessary before we can expect to see a trend away from the use of viral vectors. Viral vectors are still considered the primary choice for gene delivery, evident by their use in 67% of current clinical trials versus less than 1% of non-viral vectors [124]. For novel nanomaterials, techniques and gene delivery approaches continue to be developed, the key challenge will be to balance transfection efficiency, targeting specificity, particle size, biodegradability, and cytotoxicity, as well as their short- and long-term fates in the environment. This will typically require a mechanism to prevent the removal of vectors by the reticulo-endothelial system (RES). Many nanomaterials can avoid the RES system by shielding their charged groups with PEG, proteins or polysaccharides, as highlighted with a number of examples. Additionally, the smaller the nanoparticles are, the lower the probability of surface neutralization by serum proteins and subsequent RES clearance. Quantum dots are of special interest in this respect. The co-administration of two different nanomaterials to target and deliver gene payloads to create a synergistic effect [120] and the use of theranostic nanoparticles are especially interesting developments that show great promise.

Furthermore, the use of standardized assays that can be used to determine the efficacy and fate of a variety of gene delivery vectors would enable head-to-head comparisons. Following such additional research, and detailed characterization of their interaction with the patient host, it is likely that some nanomaterials will be approved for use in humans in the near future, thus expanding the therapeutic repertoire for gene therapy.

## References

[1] Aruna K., Rao K.R., Parhana P. (2015). A Systematic Review on Nanomaterials: Properties, Synthesis and Applications. I-Manager J. Future Eng. Technol..

[2] Biju V. (2014). Chemical modifications and bioconjugate reactions of nanomaterials for sensing, imaging, drug delivery and therapy. Chem. Soc. Rev..

[3] Ylä-Herttuala S. (2012). Endgame: Glybera finally recommended for approval as the first gene therapy drug in the European Union. Mol. Ther..

[4] Giacca M., Zacchigna S. (2012). Virus-mediated gene delivery for human gene therapy. J. Control. Release.

[5] Ibraheem D., Elaissari A., Fessi H. (2014). Gene therapy and DNA delivery systems. Int. J. Pharm..

[6] Samulski R.J., Zhu X., Xiao X., Brook J.D., Housman D.E., Epstein N., Hunter L.A. (1991). Targeted integration of adeno-associated virus (AAV) into human chromosome 19. EMBO J..

[7] Kotin R.M., Linden R.M., Berns K.I. (1992). Characterization of a preferred site on human chromosome 19q for integration of adeno-associated virus DNA by non-homologous recombination. EMBO J..

[8] Nayak S., Herzog R.W. (2010). Progress and prospects: Immune responses to viral vectors. Gene Ther..

[9] Parhiz H., Shier W.T., Ramezani M. (2013). From rationally designed polymeric and peptidic systems to sophisticated gene delivery nano-vectors. Int. J. Pharm..

[10] Mellott A.J., Forrest M.L., Detamore M.S. (2013). Physical non-viral gene delivery methods for tissue engineering. Ann. Biomed. Eng..

[11] Jin L., Zeng X., Liu M., Deng Y., He N. (2014). Current progress in gene delivery technology based on chemical methods and nano-carriers. Theranostics.

[12] Raemdonck K., Vandenbroucke R.E., Demeester J., Sanders N.N., De Smedt S.C. (2008). Maintaining the silence: Reflections on long-term RNAi. Drug Discov. Today.

[13] Luzio J.P., Pryor P.R., Bright N.A. (2007). Lysosomes: Fusion and function. Nat. Rev. Mol. Cell Biol..

[14] Gu F., Crump C., Thomas G. (2001). Trans-Golgi network sorting. Cell. Mol. Life Sci..

[15] Maxfield F.R., McGraw T.E. (2004). Endocytic recycling. Nat. Rev. Mol. Cell Biol..

[16] Tomari Y., Zamore P.D. (2005). Perspective: Machines for RNAi. Genes Dev..

[17] Hamilton A.J., Baulcombe D.C. (1999). A species of small antisense RNA in posttranscriptional gene silencing in plants. Science.

[18] Elbashir S.M., Harborth J., Lendeckel W., Yalcin A., Weber K., Tuschl T. (2001). Duplexes of 21-nucleotide RNAs mediate RNA interference in cultured mammalian cells. Nature.

[19] Sattler K.D. (2010). Handbook of Nanophysics: Nanomedicine and Nanorobotics.

[20] Erathodiyil N., Ying J.Y. (2011). Functionalization of inorganic nanoparticles for bioimaging applications. Acc. Chem. Res..

[21] Son S., Nam J., Kim J., Kim S., Kim W.J. (2014). i-Motif-driven Au nanomachines in programmed siRNA delivery for gene-silencing and photothermal ablation. ACS Nano.

[22] Peng L., Huang Y., Zhang C., Niu J., Chen Y., Chu Y., Jiang Z., Gao J., Mao Z. (2016). Integration of antimicrobial peptides with gold nanoparticles as unique non-viral vectors for gene delivery to mesenchymal stem cells with antibacterial activity. Biomaterials.

[23] Wagstaff K.M., Jans D.A. (2006). Protein transduction: Cell penetrating peptides and their therapeutic applications. Curr. Med. Chem..

[24] Ziegler A., Nervi P., Dürrenberger M., Seelig J. (2005). The cationic cell-penetrating peptide CPPTAT derived from the HIV-1 protein TAT is rapidly transported into living fibroblasts: Optical, biophysical, and metabolic evidence. Biochemistry.

[25] Peng L., Niu J., Zhang C., Yu W., Wu J., Shan Y., Wang X., Shen Y., Mao Z., Liang W. (2014). TAT conjugated cationic noble metal nanoparticles for gene delivery to epidermal stem cells. Biomaterials.

[26] Lakshmipathy U., Pelacho B., Sudo K., Linehan J.L., Coucouvanis E., Kaufman D.S., Verfaillie C.M. (2004). Efficient transfection of embryonic and adult stem cells. Stem Cells.

[27] Domashenko A., Gupta S., Cotsarelis G. (2000). Efficient delivery of transgenes to human hair follicle progenitor cells using topical lipoplex. Nat. Biotechnol..

[28] Lu C.H., Yang H.H., Zhu C.L., Chen X., Chen G.N. (2009). A graphene platform for sensing biomolecules. Angew. Chem..

[29] Yang X., Zhang X., Liu Z., Ma Y., Huang Y., Chen Y. (2008). High-efficiency loading and controlled release of doxorubicin hydrochloride on graphene oxide. J. Phys. Chem. C.

[30] Lu C., Zhu C., Li J., Liu J., Chen X., Yang H. (2010). Using graphene to protect DNA from cleavage during cellular delivery. Chem. Commun..

[31] Kim H., Kim W.J. (2014). Photothermally controlled gene delivery by reduced graphene oxide–polyethylenimine nanocomposite. Small.

[32] Zhi F., Dong H., Jia X., Guo W., Lu H., Yang Y., Ju H., Zhang X., Hu Y. (2013). Functionalized graphene oxide mediated adriamycin delivery and miR-21 gene silencing to overcome tumor multidrug resistance in vitro. PLoS ONE.

[33] Murray C.B., Kagan C., Bawendi M. (2000). Synthesis and characterization of monodisperse nanocrystals and close-packed nanocrystal assemblies. Annu. Rev. Mater. Sci..

[34] Kortshagen U. (2009). Nonthermal plasma synthesis of semiconductor nanocrystals. J. Phys. D.

[35] Wu P., Yan X. (2013). Doped quantum dots for chemo/biosensing and bioimaging. Chem. Soc. Rev..

[36] Yang H.N., Park J.S., Jeon S.Y., Park W., Na K., Park K. (2014). The effect of quantum dot size and poly(ethylenimine) coating on the efficiency of gene delivery into human mesenchymal stem cells. Biomaterials.

[37] Xie J., Lee S., Chen X. (2010). Nanoparticle-based theranostic agents. Adv. Drug Deliv. Rev..

[38] Tomitaka A., Jeun M., Bae S., Takemura Y. (2011). Evaluation of magnetic and thermal properties of ferrite nanoparticles for biomedical applications. J. Magn..

[39] Kami D., Kitani T., Kishida T., Mazda O., Toyoda M., Tomitaka A., Ota S., Ishii R., Takemura Y., Watanabe M. (2014). Pleiotropic functions of magnetic nanoparticles for ex vivo gene transfer. Nanomed. Nanotechnol. Biol. Med..

[40] Mahmoudi M., Sant S., Wang B., Laurent S., Sen T. (2011). Superparamagnetic iron oxide nanoparticles (SPIONs): Development, surface modification and applications in chemotherapy. Adv. Drug Deliv. Rev..

[41] Veiseh O., Kievit F.M., Liu V., Fang C., Stephen Z.R., Ellenbogen R.G., Zhang M. (2013). In vivo safety evaluation of polyarginine coated magnetic nanovectors. Mol. Pharm..

[42] Li D., Tang X., Pulli B., Lin C., Zhao P., Cheng J., Lv Z., Yuan X., Luo Q., Cai H. (2014). Theranostic nanoparticles based on bioreducible polyethylenimine-coated iron oxide for reduction-responsive gene delivery and magnetic resonance imaging. Int. J. Nanomed..

[43] Voronina N., Lemcke H., Wiekhorst F., Kühn J., Rimmbach C., Steinhoff G., David R. (2016). Non-viral magnetic engineering of endothelial cells with microRNA and plasmid-DNA—An optimized targeting approach. Nanomed. Nanotechnol. Biol. Med..

[44] Iijima S. (1991). Helical microtubules of graphitic carbon. Nature.

[45] Iijima S. (2002). Carbon nanotubes: Past, present, and future. Phys. B.

[46] Iijima S., Ichihashi T. (1993). Single-shell carbon nanotubes of 1-nm diameter. Nature.

[47] Liu Z., Tabakman S., Welsher K., Dai H. (2009). Carbon nanotubes in biology and medicine: In vitro and in vivo detection, imaging and drug delivery. Nano. Res..

[48] Niyogi S., Hamon M., Hu H., Zhao B., Bhowmik P., Sen R., Itkis M., Haddon R. (2002). Chemistry of single-walled carbon nanotubes. Acc. Chem. Res..

[49] Zheng D., Ye J., Zhang W. (2008). Some properties of sodium dodecyl sulfate functionalized multiwalled carbon nanotubes electrode and its application on detection of dopamine in the presence of ascorbic acid. Electroanalysis.

[50] Chen R.J., Zhang Y., Wang D., Dai H. (2001). Noncovalent sidewall functionalization of single-walled carbon nanotubes for protein immobilization. J. Am. Chem. Soc..

[51] Georgakilas V., Kordatos K., Prato M., Guldi D.M., Holzinger M., Hirsch A. (2002). Organic functionalization of carbon nanotubes. J. Am. Chem. Soc..

[52] Chen J., Liu H., Weimer W.A., Halls M.D., Waldeck D.H., Walker G.C. (2002). Noncovalent engineering of carbon nanotube surfaces by rigid, functional conjugated polymers. J. Am. Chem. Soc..

[53] Zhao J., Lu J.P., Han J., Yang C. (2003). Noncovalent functionalization of carbon nanotubes by aromatic organic molecules. Appl. Phys. Lett..

[54] Nakayama-Ratchford N., Bangsaruntip S., Sun X., Welsher K., Dai H. (2007). Noncovalent functionalization of carbon nanotubes by fluorescein—Polyethylene glycol: Supramolecular conjugates with pH-dependent absorbance and fluorescence. J. Am. Chem. Soc..

[55] Wang L., Shi J., Zhang H., Li H., Gao Y., Wang Z., Wang H., Li L., Zhang C., Chen C. (2013). Synergistic anticancer effect of RNAi and photothermal therapy mediated by functionalized single-walled carbon nanotubes. Biomaterials.

[56] O’Connell M.J., Bachilo S.M., Huffman C.B., Moore V.C., Strano M.S., Haroz E.H., Rialon K.L., Boul P.J., Noon W.H., Kittrell C. (2002). Band gap fluorescence from individual single-walled carbon nanotubes. Science.

[57] Zhou F., Wu S., Song S., Chen W.R., Resasco D.E., Xing D. (2012). Antitumor immunologically modified carbon nanotubes for photothermal therapy. Biomaterials.

[58] Florea B.I., Meaney C., Junginger H.E., Borchard G. (2002). Transfection efficiency and toxicity of polyethylenimine in differentiated Calu-3 and nondifferentiated COS-1 cell cultures. AAPS J..

[59] Akinc A., Thomas M., Klibanov A.M., Langer R. (2005). Exploring polyethylenimine-mediated DNA transfection and the proton sponge hypothesis. J. Gene Med..

[60] Behnam B., Shier W.T., Nia A.H., Abnous K., Ramezani M. (2013). Non-covalent functionalization of single-walled carbon nanotubes with modified polyethyleneimines for efficient gene delivery. Int. J. Pharm..

[61] Kong F., Liu F., Li W., Guo X., Wang Z., Zhang H., Li Q., Luo L., Du Y., Jin Y. (2016). Smart carbon nanotubes with laser-Controlled behavior in gene delivery and therapy through a non-Digestive trafficking pathway. Small.

[62] Siu K.S., Chen D., Zheng X., Zhang X., Johnston N., Liu Y., Yuan K., Koropatnick J., Gillies E.R., Min W. (2014). Non-covalently functionalized single-walled carbon nanotube for topical siRNA delivery into melanoma. Biomaterials.

[63] Liu X., Zhang Y., Ma D., Tang H., Tan L., Xie Q., Yao S. (2013). Biocompatible multi-walled carbon nanotube-chitosan–folic acid nanoparticle hybrids as GFP gene delivery materials. Colloids Surfaces B.

[64] Geyik C., Evran S., Timur S., Telefoncu A. (2014). The covalent bioconjugate of multiwalled carbon nanotube and amino-modified linearized plasmid DNA for gene delivery. Biotechnol. Prog..

[65] Jain S., Thanki K., Pandi N.K., Kushwah V. (2016). Estradiol functionalized multi-walled carbon nanotubes as renovated strategy for efficient gene delivery. RSC Adv..

[66] Lohcharoenkal W., Wang L., Chen Y.C., Rojanasakul Y. (2014). Protein nanoparticles as drug delivery carriers for cancer therapy. Biomed. Res. Int..

[67] Jahanshahi M., Babaei Z. (2008). Protein nanoparticle: A unique system as drug delivery vehicles. Afr. J. Biotechnol..

[68] Kommareddy S., Shenoy D.B., Amiji M.M. (2005). Gelatin nanoparticles and their biofunctionalization. Nanotechnol. Life Sci..

[69] Lee S.J., Yhee J.Y., Kim S.H., Kwon I.C., Kim K. (2013). Biocompatible gelatin nanoparticles for tumor-targeted delivery of polymerized siRNA in tumor-bearing mice. J. Control. Release.

[70] Moran M., Rosell N., Ruano G., Busquets M., Vinardell M. (2015). Gelatin-based nanoparticles as DNA delivery systems: Synthesis, physicochemical and biocompatible characterization. Coll. Surfaces B.

[71] Farrugia C.A., Groves M.J. (1999). Gelatin behaviour in dilute aqueous solution: Designing a nanoparticulate formulation. J. Pharm. Pharmacol..

[72] Andersson C., Iresjö B., Lundholm K. (1991). Identification of tissue sites for increased albumin degradation in sarcoma-bearing mice. J. Surg. Res..

[73] Matsumura Y., Maeda H. (1986). A new concept for macromolecular therapeutics in cancer chemotherapy: Mechanism of tumoritropic accumulation of proteins and the antitumor agent smancs. Cancer Res..

[74] Karimi M., Avci P., Mobasseri R., Hamblin M.R., Naderi-Manesh H. (2013). The novel albumin–chitosan core–shell nanoparticles for gene delivery: Preparation, optimization and cell uptake investigation. J. Nanopart. Res..

[75] Han J., Wang Q., Zhang Z., Gong T., Sun X. (2014). Cationic bovine serum albumin based self-assembled nanoparticles as siRNA delivery vector for treating lung metastatic cancer. Small.

[76] Altman G.H., Diaz F., Jakuba C., Calabro T., Horan R.L., Chen J., Lu H., Richmond J., Kaplan D.L. (2003). Silk-based biomaterials. Biomaterials.

[77] Shchepelina O., Drachuk I., Gupta M.K., Lin J., Tsukruk V.V. (2011). Silk-on-silk layer-by-layer microcapsules. Adv. Mater..

[78] Li L., Puhl S., Meinel L., Germershaus O. (2014). Silk fibroin layer-by-layer microcapsules for localized gene delivery. Biomaterials.

[79] Shukla R., Cheryan M. (2001). Zein: The industrial protein from corn. Ind. Crops Prod..

[80] Regier M.C., Taylor J.D., Borcyk T., Yang Y., Pannier A.K. (2012). Fabrication and characterization of DNA-loaded zein nanospheres. J. Nanobiotechnol..

[81] Karthikeyan K., Krishnaswamy V.R., Lakra R., Kiran M., Korrapati P.S. (2015). Fabrication of electrospun zein nanofibers for the sustained delivery of siRNA. J. Mater. Sci. Mater. Med..

[82] Urry D.W. (1997). Physical chemistry of biological free energy transduction as demonstrated by elastic protein-based polymers. J. Phys. Chem. B.

[83] Dash B.C., Thomas D., Monaghan M., Carroll O., Chen X., Woodhouse K., O’Brien T., Pandit A. (2015). An injectable elastin-based gene delivery platform for dose-dependent modulation of angiogenesis and inflammation for critical limb ischemia. Biomaterials.

[84] Dash B.C., Mahor S., Carroll O., Mathew A., Wang W., Woodhouse K.A., Pandit A. (2011). Tunable elastin-like polypeptide hollow sphere as a high payload and controlled delivery gene depot. J. Control. Release.

[85] Lee H.J., Bae Y. (2011). Cross-linked nanoassemblies from poly(ethylene glycol)-poly(aspartate) block copolymers as stable supramolecular templates for particulate drug delivery. Biomacromolecules..

[86] Dou X., Hu Y., Zhao N., Xu F. (2014). Different types of degradable vectors from low-molecular-weight polycation-functionalized poly(aspartic acid) for efficient gene delivery. Biomaterials.

[87] Xu F., Yang W. (2011). Polymer vectors via controlled/living radical polymerization for gene delivery. Prog. Polym. Sci..

[88] Unzueta U., Saccardo P., Domingo-Espín J., Cedano J., Conchillo-Solé O., García-Fruitós E., Céspedes M.V., Corchero J.L., Daura X., Mangues R. (2014). Sheltering DNA in self-organizing, protein-only nano-shells as artificial viruses for gene delivery. Nanomed. Nanotechnol. Biol. Med..

[89] Felgner P.L., Gadek T.R., Holm M., Roman R., Chan H.W., Wenz M., Northrop J.P., Ringold G.M., Danielsen M. (1987). Lipofection: A highly efficient, lipid-mediated DNA-transfection procedure. Proc. Natl. Acad. Sci. USA.

[90] Radler J.O., Koltover I., Salditt T., Safinya C.R. (1997). Structure of DNA-cationic liposome complexes: DNA intercalation in multilamellar membranes in distinct interhelical packing regimes. Science.

[91] De Ilarduya C.T., Sun Y., Düzgüneş N. (2010). Gene delivery by lipoplexes and polyplexes. Eur. J. Pharm. Sci..

[92] Khatri N., Baradia D., Vhora I., Rathi M., Misra A. (2014). cRGD grafted liposomes containing inorganic nano-precipitate complexed siRNA for intracellular delivery in cancer cells. J. Control. Release.

[93] Byrne J.D., Betancourt T., Brannon-Peppas L. (2008). Active targeting schemes for nanoparticle systems in cancer therapeutics. Adv. Drug Deliv. Rev..

[94] Kullberg M., McCarthy R., Anchordoquy T.J. (2014). Gene delivery to Her-2 breast cancer cells using a two-component delivery system to achieve specificity. Nanomed. Nanotechnol. Biol. Med..

[95] Benz C.C., Scott G.K., Sarup J.C., Johnson R.M., Tripathy D., Coronado E., Shepard H.M., Osborne C.K. (1992). Estrogen-dependent, tamoxifen-resistant tumorigenic growth of MCF-7 cells transfected with HER2/neu. Breast Cancer Res. Treat..

[96] Whitehead K.A., Dorkin J.R., Vegas A.J., Chang P.H., Veiseh O., Matthews J., Fenton O.S., Zhang Y., Olejnik K.T., Yesilyurt V. (2014). Degradable lipid nanoparticles with predictable in vivo siRNA delivery activity. Nat. Commun..

[97] Goldberg M., Howard K.A. (2013). Lipidoids: A combinatorial approach to siRNA delivery. RNA Interference from Biology to Therapeutics.

[98] Knapp C.M., He J., Lister J., Whitehead K.A. (2016). Lipidoid nanoparticle mediated silencing of Mcl-1 induces apoptosis in mantle cell lymphoma. Exp. Biol. Med..

[99] Moon J., Lee S., Han S., Kim E., Cho H., Lee W., Kim M., Kim T., Park H., Rhee J. (2016). Inhibition of hepatitis C virus in mouse models by lipidoid nanoparticle-mediated systemic delivery of siRNA against PRK2. Nanomed. Nanotechnol. Biol. Med..

[100] Akinc A., Zumbuehl A., Goldberg M., Leshchiner E.S., Busini V., Hossain N., Bacallado S.A., Nguyen D.N., Fuller J., Alvarez R. (2008). A combinatorial library of lipid-like materials for delivery of RNAi therapeutics. Nat. Biotechnol..

[101] Hait S., Moulik S. (2002). Gemini surfactants: A distinct class of self-assembling molecules. Curr. Sci.-Bangalore.

[102] Wettig S.D., Verrall R.E., Foldvari M. (2008). Gemini surfactants: A new family of building blocks for non-viral gene delivery systems. Curr. Gene Ther..

[103] Alqawlaq S., Sivak J.M., Huzil J.T., Ivanova M.V., Flanagan J.G., Beazely M.A., Foldvari M. (2014). Preclinical development and ocular biodistribution of gemini-DNA nanoparticles after intravitreal and topical administration: Towards non-invasive glaucoma gene therapy. Nanomed. Nanotechnol. Biol. Med..

[104] Liang W., Lam J.K. (2012). Endosomal Escape Pathways for Non-Viral Nucleic Acid Delivery Systems.

[105] Dash P., Read M., Barrett L., Wolfert M., Seymour L. (1999). Factors affecting blood clearance and in vivo distribution of polyelectrolyte complexes for gene delivery. Gene Ther..

[106] Glodde M., Sirsi S.R., Lutz G.J. (2006). Physiochemical properties of low and high molecular weight poly(ethylene glycol)-grafted poly(ethylene imine) copolymers and their complexes with oligonucleotides. Biomacromolecules.

[107] Chen C.K., Law W.C., Aalinkeel R., Nair B., Kopwitthaya A., Mahajan S.D., Reynolds J.L., Zou J., Schwartz S.A., Prasad P.N. (2012). Well-defined degradable cationic polylactide as nanocarrier for the delivery of siRNA to silence angiogenesis in prostate cancer. Adv. Healthcare Mater..

[108] Chen C., Jones C.H., Mistriotis P., Yu Y., Ma X., Ravikrishnan A., Jiang M., Andreadis S.T., Pfeifer B.A., Cheng C. (2013). Poly(ethylene glycol)-block-cationic polylactide nanocomplexes of differing charge density for gene delivery. Omaterials.

[109] Wang Y., Zheng M., Meng F., Zhang J., Peng R., Zhong Z. (2011). Branched polyethylenimine derivatives with reductively cleavable periphery for safe and efficient in vitro gene transfer. Biomacromolecules.

[110] He Y., Cheng G., Xie L., Nie Y., He B., Gu Z. (2013). Polyethyleneimine/DNA polyplexes with reduction-sensitive hyaluronic acid derivatives shielding for targeted gene delivery. Biomaterials.

[111] Jiang G., Park K., Kim J., Kim K.S., Hahn S.K. (2009). Target specific intracellular delivery of siRNA/PEI—HA complex by receptor mediated endocytosis. Mol. Pharm..

[112] Chen H., Liu X., Dou Y., He B., Liu L., Wei Z., Li J., Wang C., Mao C., Zhang J. (2013). A pH-responsive cyclodextrin-based hybrid nanosystem as a nonviral vector for gene delivery. Biomaterials.

[113] Zhang J., Li X., Li X. (2012). Stimuli-triggered structural engineering of synthetic and biological polymeric assemblies. Prog. Polym. Sci..

[114] Wenz G. (1994). Cyclodextrins as building blocks for supramolecular structures and functional units. Angew. Chem. Int. Ed. Engl..

[115] Cryan S., Holohan A., Donohue R., Darcy R., O’Driscoll C.M. (2004). Cell transfection with polycationic cyclodextrin vectors. Eur. J. Pharm. Sci..

[116] Godinho B.M., Ogier J.R., Quinlan A., Darcy R., Griffin B.T., Cryan J.F., O’Driscoll C.M. (2014). PEGylated cyclodextrins as novel siRNA nanosystems: Correlations between polyethylene glycol length and nanoparticle stability. Int. J. Pharm..

[117] Evans J.C., Malhotra M., Guo J., O’Shea J.P., Hanrahan K., O’Neill A., Landry W.D., Griffin B.T., Darcy R., Watson R.W. (2016). Folate-targeted amphiphilic cyclodextrin. siRNA nanoparticles for prostate cancer therapy exhibit PSMA mediated uptake, therapeutic gene silencing in vitro and prolonged circulation in vivo. Nanomed. Nanotechnol. Biol. Med..

[118] Hattori Y., Maitani Y. (2004). Enhanced in vitro DNA transfection efficiency by novel folate-linked nanoparticles in human prostate cancer and oral cancer. J. Control. Release.

[119] Merdan T., Kopec̆ek J., Kissel T. (2002). Prospects for cationic polymers in gene and oligonucleotide therapy against cancer. Adv. Drug Deliv. Rev..

[120] Zeng L., Li J., Wang Y., Qian C., Chen Y., Zhang Q., Wu W., Lin Z., Liang J., Shuai X. (2014). Combination of siRNA-directed Kras. oncogene silencing and arsenic-induced apoptosis using a nanomedicine strategy for the effective treatment of pancreatic cancer. Nanomed. Nanotechnol. Biol. Med..

[121] Ralph J., Lundquist K., Brunow G., Lu F., Kim H., Schatz P.F., Marita J.M., Hatfield R.D., Ralph S.A., Christensen J.H. (2004). Lignins: Natural polymers from oxidative coupling of 4-hydroxyphenyl-propanoids. Phytochem. Rev..

[122] Caicedo H.M., Dempere L.A., Vermerris W. (2012). Template-mediated synthesis and bio-functionalization of flexible lignin-based nanotubes and nanowires. Nanotechnology.

[123] Ten E., Ling C., Wang Y., Srivastava A., Dempere L.A., Vermerris W. (2013). Lignin nanotubes as vehicles for gene delivery into human cells. Biomacromolecules.

[124] Ginn S.L., Alexander I.E., Edelstein M.L., Abedi M.R., Wixon J. (2013). Gene therapy clinical trials worldwide to 2012–an update. J. Gene Med..

